# Association of serum secreted protein acidic and rich in cysteine-like protein 1 with metabolic measures and dyslipidemia among Chinese adults

**DOI:** 10.3389/fendo.2022.1018657

**Published:** 2022-10-27

**Authors:** Chunyan Hu, Shuangyuan Wang, Lin Lin, Hongyan Qi, Hong Lin, Xiaojing Jia, Yuanyue Zhu, Xueyan Wu, Mian Li, Tiange Wang, Zhiyun Zhao, Min Xu, Yu Xu, Weiqing Wang, Guang Ning, Yufang Bi, Donghui Li, Yuhong Chen, Meng Dai, Jieli Lu

**Affiliations:** ^1^ Department of Endocrine and Metabolic Diseases, Shanghai Institute of Endocrine and Metabolic Diseases, Ruijin Hospital, Shanghai Jiao Tong University School of Medicine, Shanghai, China; ^2^ Shanghai National Clinical Research Center for Metabolic Diseases, Key Laboratory for Endocrine and Metabolic Diseases of the National Health Commission of the PR China, Shanghai National Center for Translational Medicine, Ruijin Hospital, Shanghai Jiao Tong University School of Medicine, Shanghai, China; ^3^ Department of Gastrointestinal Medical Oncology, The University of Texas MD Anderson Cancer Center, Houston, TX, United States

**Keywords:** Sparcl1, metabolic disease, lipid, dyslipidemia, triglyceride

## Abstract

**Objectives:**

Recent studies found that secreted protein acidic and rich in cysteine-like protein 1 (Sparcl1) could inhibit lipid droplets accumulation by peroxisome proliferator-activated receptor-gamma (PPARγ) signal pathway. However, the associations of serum Sparcl1 level with lipids profiles and other metabolic phenotypes remain unknown in human population study.

**Methods:**

We determined serum Sparcl1 using sandwich enzyme-linked immunosorbent assays among 1750 adults aged 40 years and older from a community in Shanghai, China. Generalized linear regression models were used to evaluate the association between Sparcl1 and metabolic measures. Multivariable-adjusted logistic regression analyses were performed to evaluate the relationship of serum Sparcl1 with prevalent dyslipidemia.

**Results:**

With the increment of serum Sparcl1, participants tended to have lower level of triglycerides, and higher level of high-density lipoprotein cholesterol (all P for trend < 0.01). No significant associations between serum Sparcl1 and glucose, blood pressure, or body size were observed. The generalized linear regression models suggested that per standard deviation (SD) increment of serum Sparcl1 was significantly inversely associated with triglycerides (β= -0.06, P=0.02). The prevalence of dyslipidemia decreased across the sparcl1 quartiles (P for trend <0.01). After controlling the potential confounders, participants in the highest quartile of sparcl1 concentration had the lowest prevalence of dyslipidemia (odds ratio [OR], 0.69; 95% confidence interval [CI], 0.52-0.91), compared with the lowest quartile. Per SD increment of Sparcl1 was associated with 20% (OR, 0.80; 95%CI, 0.69-0.94) lower prevalence of hypertriglyceridemia and 12% (OR, 0.88; 95%CI, 0.79-0.97) lower prevalence of dyslipidemia. The association between serum Sparcl1 and dyslipidemia were generally consistent across subgroups (all P for interaction > 0.05).

**Conclusion:**

Serum Sparcl1 was significantly associated with decreased risk of prevalent dyslipidemia in Chinese population. Further studies are warranted to confirm this association.

## Introduction

Secreted protein acidic and rich in cysteine like protein 1 (Sparcl1), belonging to the SPARC protein family, is a secreted glycoprotein that was found to be involved in the regulation of many physiological processes, including cell adhesion, migration, and proliferation (1, 2). Sparcl1 was recognized as a major player in the regulation of tumor biology (1–3). In addition, Sparcl1 was also reported to be involved in the development of Alzheimer’s disease and ischemic stroke (4–6).

Recently, Sparcl1 was reported to have a negative effect during preadipocyte differentiation and inhibits lipid droplet accumulation through regulating peroxisome proliferator-activated receptor-gamma (PPARγ), lipoprotein lipase (LPL) and insulin-like growth factor 1 (7), suggesting that Sparcl1 might play an important role in the development of metabolic disease. However, evidence on the association of Sparcl1 with systematic metabolic profiles and dyslipidemia in human population is unknown.

Thus, to reveal whether serum Sparcl1 is associated with metabolic phenotypes in human population, we systematically examined the levels of serum Sparcl1, metabolic measures, particularly, lipid profiles and dyslipidemia in a cross-sectional study among Chinese population.

## Materials and methods

### Study population

The study participants were from a community cohort in Shanghai, China. Details of the cohort could be found in previous studies (8, 9). Briefly, during March and August in 2010, a total of 10 375 adults aged 40 years or older were recruited. The exclusion criteria of the current study includes: 1) with missing data on biochemical indicators including glucose measurements, insulin and lipids; 2) diagnosed with malignant tumor, stroke, myocardial infarction and coronary heart disease; 3) with thyroid disease; 4) using glucose-lowering drugs, lipid-lowering drugs, antihypertensive drugs, glucocorticoids, thyroid hormones and levothyroxine; 5) with previous liver disease including viral hepatitis, autoimmune liver disease, cirrhosis and liver cancer; 6) with excessive alcohol consumption: ≥140 g/week for male or ≥70 g/week for female; 7) with ≥ 3 times the upper limit of normal serum alanine aminotransferase and aspartate aminotransferase. Of the remaining 3181 candidates, 1750 eligible participants with complete data were randomly selected for the current study.

The study protocol was approved by the Ethics Committee of Ruijin Hospital, Shanghai Jiao-Tong University School of Medicine. All participates signed written informed consent and took a comprehensive physical examination and interview.

### Data collection

Using standardized questionnaires, we collected detailed information on sociodemographic characteristics, lifestyle habits, and medical history. Current smokers or drinkers were defined as smoking cigarettes or consuming alcohol regularly in the past 6 months. Physically active was defined as moderate intensity physical activity ≥150 minutes per week, or vigorous intensity aerobic physical activity ≥75 minutes per week, or a combination of moderate and vigorous intensity activity ≥150 minutes per week. Height and weight were measured with participants wearing lightweight clothes and no shoes. Body mass index (BMI) was calculated as body weight in kilograms divided by height squared in meters. Waist circumference was measured using a non-stretch tape at the umbilical level in a standing position with normal breathing. After at least 5-min rest, blood pressure was measured using an automated electronic device (OMRON Model HEM-725 FUZZY, Omron Company, Dalian, China) at a non-dominant arm. The average of three measurements was used to analysis.

All participants were asked to keep fasting overnight at least 10 hours. A standard 75-g oral glucose tolerance test were conducted. Fasting plasma glucose (FPG) and 2-hour postload plasma glucose (2h-PG) levels were measured using the glucose oxidase method with an autoanalyzer (Modular P800, Roche, Basel, Switzerland). Hemoglobin A1c (HbA1c) was measured using high-performance liquid chromatography (D-10; Bio-Rad, Hercules, CA, USA) in the central laboratory located at Shanghai Institute of Endocrine and Metabolic Diseases, which is certified by the College of American Pathologists. Serum insulin was measured using an electrochemiluminescence assay (Modular E170, Roche, Basel, Switzerland). The homoeostasis model assessment of insulin resistance (HOMA-IR) index was calculated as fasting insulin (mIU/mL)×fasting glucose (mmol/L)/22.5 (10). HOMA of β-cell function (HOMA-B) was calculated as [20×fasting insulin (mIU/mL)]/[fasting glucose (mmol/L)-3.5] (10). Serum triglyceride (TG), total cholesterol (TC), low-density lipoprotein cholesterol (LDL-C), and high-density lipoprotein cholesterol (HDL-C) were measured on the autoanalyzer (Modular Analytics P800 and Modular E170; Roche, Basel, Switzerland).

### Enzyme-linked immunosorbent assay for serum Sparcl1 level

Blood samples from all participants were centrifuged at 4°C after collected, and serum were aliquoted to separate tubes and stored at –80°C, without repeated freezing and thawing. As described previously (11), serum Sparcl1 was determined using sandwich enzyme-linked immunosorbent assays (R&D, DY2728). The intra-assay and inter-assay coefficients of variations for serum Sparcl1 were 5.6% and 4.4%, respectively.

### Definitions

Dyslipidemia was defined using the National Cholesterol Education Program Adult Treatment Panel III criteria (12). Participants with any of the following conditions were defined as having dyslipidemia: (a) TC ≥ 6.22 mmol/L; (b) LDL-C ≥ 4.14 mmol/L; (c) TG ≥ 2.26 mmol/L; (d) HDL-C < 1.04 mmol/L (12).

### Statistical analysis

Continuous variables were presented as mean ± standard deviation (SD) or median (inter-quartile range) and categorical variables were presented as number (proportion). P for trend according to quartiles of serum Sparcl1 was performed using linear regression for continuous variables and χ2 testing for categorical variables.

The generalized linear regression models adjusting for age, sex, education of high-school or above, smoking and drinking status, physically active and BMI (except for the analysis of BMI) were used to evaluate the association between per SD increment of serum Sparcl1 and metabolic measurements (including BMI, waist circumference, FPG, 2h-PG, HbA1c, HOMA-IR, HOMA-B, systolic blood pressure, diastolic blood pressure, TC, TG, HDL-C, LDL-C).

Potential non-linear association between the level of serum Sparcl1 and prevalence of dyslipidemia was examined using restricted cubic splines (13). Three knots at the 5th, 50th and 95th percentiles for the level of serum Sparcl1 were used in the analysis. ORs (95% CIs) were adjusted for age, sex, education of high-school or above, smoking and drinking status, physically active and BMI. Test for non-linearity was performed using likelihood ratio tests, comparing a model with only the linear term against a model containing the linear and restricted cubic spline term. If a test for non-linearity was not significant, a test for linearity was additionally conducted, comparing a model containing the linear term with a model containing only the covariates.

Multivariable adjusted logistic regression analysis was used to evaluate the association between serum Sparcl1 and prevalence of dyslipidemia and individual components. The multivariable model was adjusted for age, sex, education of high-school or above, smoking and drinking status, physical active and BMI. We also performed subgroup analysis on the association of serum Sparcl1 and dyslipidemia according to sex, age, current smoker (yes/no), high education (yes/no), physically active (yes/no) and BMI. The P values for interaction were calculated by likelihood ratio tests comparing models with and without the interaction terms.

Statistical analyses were performed using SAS (version 9.4, Cary, NC) or R (version 4.1.2). A two-sided P value<0.05 was considered as statistically significant.

## Results

### Baseline characteristics according to serum Sparcl1 level

Among the 1750 participants included, the mean (SD) age of the study participants was 55.74 ±( 8.37) years. The mean (SD) of serum Sparcl1 level was 701.36 ± (501.48) ng/mL. Baseline characteristics according to the quartiles of serum Sparcl1 were presented in **Table 1**. With the increment of serum Sparcl1, participants tended to be older, have lower level of TG, while have higher level of HDL-C (all P for trend < 0.05; **Table 1**).

**Table 1:** Baseline characteristics according to the quartiles of serum Sparcl1.

	Sparcl1 (ng/mL)				
	Quartile 1 (≤ 331.46)	Quartile 2 (331.47- 599.72)	Quartile 3 (599.73- 984.12)	Quartile 4 (≥ 984.12)	P for trend
No, %	437 (24.97)	438 (25.03)	438 (25.03)	437 (24.97)	
Age, years	55.05 ± 8.65	55.95 ± 8.55	55.56 ± 8.35	56.41 ± 7.87	0.04
Male, n (%)	120 (27.46)	125 (28.54)	119 (27.17)	111 (25.40)	0.43
Current smoker, n (%)	70 (16.02)	70 (15.98)	60 (13.70)	69 (15.79)	0.70
Current drinker, n (%)	6 (1.37)	7 (1.60)	10 (2.28)	4 (0.92)	0.79
High education, n (%)	77 (17.62)	74 (16.89)	94 (21.46)	91 (20.82)	0.09
Physically active, n (%)	65 (14.87)	59 (13.47)	58 (13.24)	62 (14.19)	0.76
BMI, kg/m^2^	25.20 ± 3.18	24.98 ± 3.27	25.07 ± 3.19	24.87 ± 3.40	0.19
WC, cm	81.63 ± 8.49	81.56 ± 8.58	81.15 ± 8.45	81.04 ± 9.14	0.24
SBP, mmHg	135.65 ± 18.16	135.35 ± 18.45	134.97 ± 17.95	134.88 ± 17.02	0.48
DBP, mmHg	81.17 ± 9.82	81.09 ± 10.09	80.97 ± 9.66	80.66 ± 9.21	0.43
FPG, mmol/L	5.38 ± 1.35	5.21 ± 1.18	5.26 ± 1.09	5.22 ± 1.12	0.09
2h-PG, mmol/L	7.50 ± 3.55	7.06 ± 2.99	7.16 ± 2.95	7.16 ± 3.24	0.18
HbA1c, %	5.71 ± 0.91	5.71 ± 0.76	5.67 ± 0.70	5.65 ± 0.64	0.21
HOMA-IR	1.59 (1.09-2.29)	1.44 (1.00-2.12)	1.52 (1.00-2.17)	1.47 (0.99-2.13)	0.05
HOMA-B	85.04 (55.15-126.67)	88.32 (56.99-122.06)	83.00 (57.14-122.64)	83.67 (55.63-122.86)	0.55
TG, mmol/L	1.37 (1.00-1.90)	1.27 (0.92-1.83)	1.26 (0.96-1.75)	1.25 (0.93-1.66)	0.009
TC, mmol/L	5.28 ± 0.93	5.24 ± 0.94	5.35 ± 1.00	5.35 ± 0.96	0.12
LDL-C, mmol/L	3.15 ± 0.81	3.12 ± 0.80	3.19 ± 0.89	3.23 ± 0.83	0.10
HDL-C, mmol/L	1.32 ± 0.32	1.33 ± 0.32	1.36 ± 0.35	1.37 ± 0.32	0.006

BMI, body mass index; WC, waist circumference; SBP, systolic blood pressure; DBP, diastolic blood pressure; FPG, fasting plasma glucose; 2h-PG, 2-hour postload plasma glucose; HbA1c, Hemoglobin A1c; HOMA-IR, the homoeostasis model assessment of insulin resistance; HOMA-B, the homoeostasis model assessment of β-cell function; TG, triglyceride; TC, total cholesterol; LDL-C, low-density lipoprotein cholesterol; HDL-C, high-density lipoprotein cholesterol.

### Association between serum Sparcl1 and metabolic profiles

We further evaluated the association between per SD increment of serum Sparcl1 and metabolic profiles using generalized linear regression models. After adjusting for age, sex, education of high-school or above, smoking and drinking status, physically active and BMI, Sparcl1 was found significantly inversely associated with TG (**Table 2**). There was no significant association between serum Sparcl1 and three glucose measurements, HOMA-IR, HOMA-B, blood pressure, waist circumference or BMI.

**Table 2:** The association between per SD increment of serum Sparcl1 levels and metabolic measurements.

	Coefficients	95%CI	P value
Body mass index	-0.11	-0.26 to 0.04	0.17
Waist circumference	0.06	-0.14 to 0.27	0.54
Fasting plasma glucose	-0.04	-0.09 to 0.02	0.21
2h-plasma glucose	-0.07	-0.22 to 0.08	0.37
HbA1c	-0.02	-0.05 to 0.02	0.33
HOMA-IR	-0.04	-0.09 to 0.02	0.18
HOMA-B	-0.65	-5.85 to 4.55	0.81
Systolic Blood Pressure	0.03	-0.74 to 0.79	0.95
Diastolic blood pressure	0.08	-0.36 to 0.51	0.73
Total cholesterol	0.02	-0.02 to 0.06	0.38
Triglycerides	-0.06	-0.11 to -0.01	0.02
Low-density lipoprotein cholesterol	0.03	-0.01 to 0.07	0.16
High-density lipoprotein cholesterol	0.01	-0.006 to 0.02	0.25

Model was adjusted for age, sex, education of high-school or above, smoking and drinking status, physically active and BMI (except for the analysis of BMI).; HbA1c, Hemoglobin A1c; HOMA-IR, the homoeostasis model assessment of insulin resistance; HOMA-B, the homoeostasis model assessment of b-cell function; BMI, body mass index.

### Association between serum Sparcl1 and dyslipidemia

**Figure 1:**
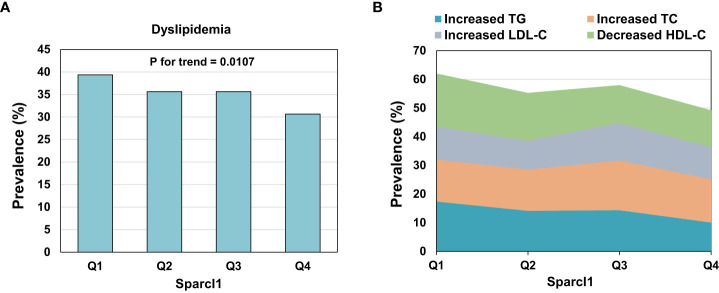
Prevalence of dyslipidemia **(A)** and individual components **(B)** according to the quartiles of serum Sparcl1. TG, triglyceride; TC, total cholesterol; LDL-C, low-density lipoprotein cholesterol; HDL-C, high-density lipoprotein cholesterol.

The prevalence of dyslipidemia was 35.31% (618/1750) in this population. Participants with dyslipidemia have lower level of serum Sparcl1 than those without dyslipidemia (725.11 vs 657.86 ng/mL, P =0.006). The prevalence of dyslipidemia (**Figure 1A**) was significantly decreased with increasing of the quartiles of serum Sparcl1 (P for trend = 0.0107). The prevalence of dyslipidemia in quartiles 1 to 4 were 39.36%, 35.62%, 35.62% and 30.66%, respectively. For individual components, only prevalence of increased TG (P for trend = 0.0034) and decreased HDL-C (P for trend = 0.0075) were significantly decreased with the increasing of the quartiles of serum Sparcl1 (**Figure 1B**).

**Figure 2:**
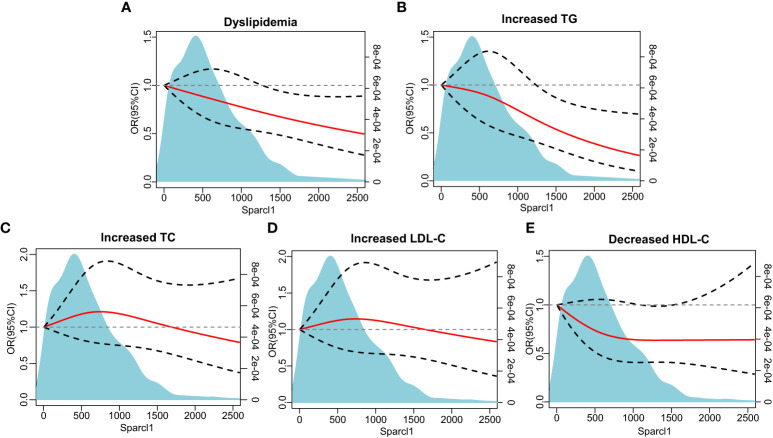
The association between serum Sparcl1 with dyslipidemia and individual components based on restricted cubic spines. **(A)** Dyslipidemia; **(B)** Increased TG; **(C)** Increased TC; **(D)** Increased LDL-C, **(E)** Decreased HDL-C. The red solid line represents a fitted relationship and black dashed lines show 95% confidence intervals. Reference line for no association (odds ratio =1.0) is indicated by grey dotted line. Area of blue represents the density distribution of serum Sparcl1. Model was adjusted for age, sex, education of high-school or above, smoking and drinking status, physically active, and body mass index. TG, triglyceride; TC, total cholesterol; LDL-C, low-density lipoprotein cholesterol; HDL-C, high-density lipoprotein cholesterol.

Multivariable-adjusted restricted cubic spline analyses indicated that there was no non-linear association between serum Sparcl1 and the prevalence of dyslipidemia (P for non-linearity = 0.93, **Figure 2A**). However, significant linear association of Sparcl1 with the prevalence of dyslipidemia were observed (P for linearity = 0.011, **Figure 2A**). For the individual components of dyslipidemia, similar trend was found in the association between serum Sparcl1 and increased TG (P for non-linearity=0.45, P for linearity = 0.0052, **Figure 2B**). No significant association was found between serum Sparcl1 and increased TC, increased LDL-C, and decreased HDL-C (all P for non-linearity > 0.05, and all P for linearity >0.05, **Figure 2**).

Logistic regression analysis suggested that serum Sparcl1 was significantly associated with decreased risk of prevalent dyslipidemia (P for trend=0.011). **Table 3**. Compared with participants with the lowest quartile of serum Sparcl1, the highest quartile of serum Sparcl1 group had a significant lower prevalence of dyslipidemia (OR, 0.68; 95%CI, 0.52-0.90, **Table 3**). Per SD increasement of serum Sparcl1 was associated with 13% (OR, 0.87; 95%CI, 0.79-0.96, P value=0.008, **Table 3**) lower frequency of dyslipidemia. Further adjustment for lifestyle factors (including smoking status, drinking status and physically activity) and BMI did not change the estimates significantly. For the components of dyslipidemia, participants in the highest quartile of serum Sparcl1 were significantly associated with lower prevalence of elevated TG (OR, 0.55; 95%CI, 0.37-0.83) in the multivariable-adjusted logistic regression model, compared with the lowest quartile group (**Table 3**). No significant associations were detected for Sparcl1 and increased TC, increased LDL-C, and decreased HDL-C.

**Table 3:** Odds ratios (95% confidence intervals) for prevalent dyslipidemia according to serum Sparcl1 levels.

	Sparcl1 (ng/mL)						
	Quartile1 (≤ 331.46)	Quartile 2 (331.47- 599.72)	Quartile 3 (599.73- 984.12)	Quartile 4 (≥ 984.12)	P for trend	Per SD increment	P value
**Dyslipidemia**							
Case/No.	172/437	156/438	156/438	134/437			
Model 1	1.00(ref)	0.85 (0.65-1.12)	0.85 (0.65-1.12)	0.68 (0.52-0.90)	0.011	0.87 (0.79-0.96)	0.008
Model 2	1.00(ref)	0.84 (0.64-1.10)	0.85 (0.64-1.12)	0.67 (0.51-0.89)	0.010	0.87 (0.79-0.96)	0.007
Model 3	1.00(ref)	0.86 (0.65-1.13)	0.86 (0.65-1.13)	0.69 (0.52-0.91)	0.014	0.88 (0.79-0.97)	0.011
**Increased TG**							
Case/No.	76/437	62/438	63/438	44/437			
Model 1	1.00(ref)	0.78 (0.54-1.13)	0.80 (0.56-1.15)	0.53 (0.36-0.79)	0.004	0.79 (0.68-0.92)	0.002
Model 2	1.00(ref)	0.78 (0.54-1.13)	0.80 (0.56-1.16)	0.54 (0.36-0.81)	0.005	0.80 (0.69-0.93)	0.003
Model 3	1.00(ref)	0.80 (0.55-1.16)	0.81 (0.56-1.17)	0.55 (0.37-0.83)	0.007	0.80 (0.69-0.94)	0.005
**Increased TC**							
Case/No.	64/437	63/438	76/438	66/437			
Model 1	1.00(ref)	0.98 (0.67-1.43)	1.22 (0.85-1.76)	1.04 (0.71-1.51)	0.57	1.00 (0.88-1.14)	0.95
Model 2	1.00(ref)	0.96 (0.66-1.40)	1.21 (0.84-1.75)	0.98 (0.67-1.43)	0.76	0.98 (0.86-1.12)	0.75
Model 3	1.00(ref)	0.97 (0.66-1.42)	1.23 (0.85-1.77)	0.99 (0.68-1.45)	0.71	0.98 (0.86-1.12)	0.79
**Increased LDL-C**							
Case/No.	50/437	44/438	57/438	49/437			
Model 1	1.00(ref)	0.86 (0.56-1.33)	1.16 (0.77-1.74)	0.98 (0.64-1.49)	0.74	1.01 (0.87-1.16)	0.95
Model 2	1.00(ref)	0.84 (0.54-1.29)	1.14 (0.76-1.72)	0.92 (0.60-1.41)	0.92	0.98 (0.84-1.13)	0.76
Model 3	1.00(ref)	0.85 (0.55-1.30)	1.17 (0.77-1.76)	0.94 (0.62-1.44)	0.83	0.98 (0.85-1.14)	0.83
**Decreased HDL-C**							
Case/No.	81/437	73/438	58/438	56/437			
Model 1	1.00(ref)	0.88 (0.62-1.25)	0.67 (0.47-0.97)	0.65 (0.45-0.94)	0.008	0.86 (0.74-0.98)	0.027
Model 2	1.00(ref)	0.87 (0.61-1.25)	0.67 (0.46-0.97)	0.67 (0.46-0.98)	0.015	0.87 (0.75-1.01)	0.061
Model 3	1.00(ref)	0.92 (0.64-1.32)	0.68 (0.46-0.99)	0.69 (0.47-1.02)	0.023	0.88 (0.76-1.02)	0.094

Model1: unadjusted; Model 2: adjusted for age and sex; Model 3: adjusted for age, sex, education of high-school or above, smoking and drinking status, physically active, and body mass index. TG, triglyceride; TC, total cholesterol; LDL-C, low-density lipoprotein cholesterol; HDL-C, high-density lipoprotein cholesterol.

**Figure 3:**
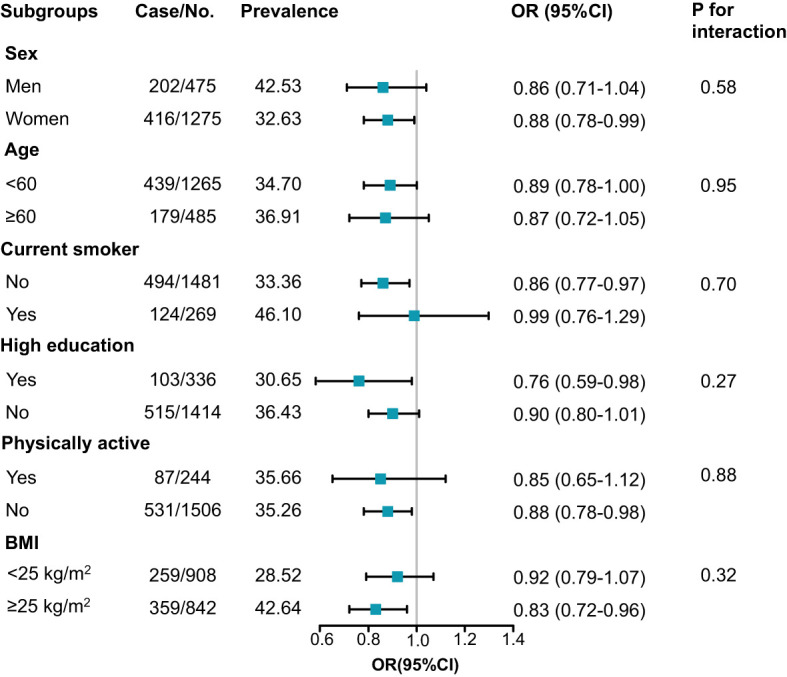
Subgroup analysis for the association between serum Sparcl1 per SD increment and prevalence of dyslipidemia. Model was adjusted for age, sex, education of high-school or above, smoking and drinking status, physically active, and BMI (except for the analysis of BMI). BMI, body mass index.

Stratified analysis for the association between per SD increment of serum Sparcl1 and dyslipidemia was presented in **Figure 3**. Inverse associations between serum Sparcl1 and dyslipidemia were generally similar across subgroups (all P for interaction > 0.05). Statistically significant association between serum Sparcl1 and dyslipidemia was observed in the subgroups of women (OR, 0.88; 95%CI, 0.78-0.99), current non-smokers (OR, 0.86; 95%CI, 0.77-0.97), high education (OR, 0.76; 95%CI, 0.59-0.98), physically inactive (OR, 0.88; 95%CI, 0.78-0.98), and BMI≥25kg/m^2^ (OR, 0.83;95% CI, 0.72-0.96).

## Discussion

This community-based cohort study found that serum Sparcl1 was inversely related to TG. Moreover, per SD increment of serum Sparcl1 was associated with 20% decreased prevalence of hypertriglyceridemia and 12% decreased prevalence of dyslipidemia. The inverse associations between serum Sparcl1 and dyslipidemia were generally similar across all subgroups. To the best of our knowledge, the current study represents the first report in population concerning the metabolic profiles and dyslipidemia in association with serum Sparcl1. These findings suggest that Sparcl1 might play an important role in the lipid metabolism and the development of dyslipidemia.

Sparcl1 was previously considered to participate in a variety of physiological functions and closely related to tumor biology (1, 3, 14). It has been found that Sparcl1 is frequently downregulated in many cancers, including colorectal carcinoma (14), prostate cancers (15), pancreatic cancer (16), etc., suggesting its potential role as a tumor suppressor (17). In addition, Sparcl1 was also found to be independently associated with ischemic stroke severity in 132 patients with acute ischemic stroke (5). The Baltimore Longitudinal Study of Aging indicated that cognitively normal minor allele carriers of *rs7695558* and *rs9998212*, which were both associated with lower brain Sparcl1 gene expression, showed accelerated memory loss or atrophy of brain volumes (6). Recently, Sparcl1 was reported to be upregulated in liver-specific Apobec1 complementation factor transgenic mice, which is charactered with a phenotype with spontaneous steatosis, fibrosis, and hepatocellular cancer (18). Sparcl1 was found to be a regulator of nonalcoholic steatohepatitis (NASH) progression (11). Mechanistic studies demonstrated that Sparcl1 is a regulator of NASH progression by promoting the expression of C-C motif chemokine ligand 2 through Toll-like receptor 4/NF-κB signaling pathway (11). Regarding the role of Sparcl1 in regulating metabolism, it was recently reported that Sparcl1 could be an inhibitory candidate for adipogenesis in mice (19). Experiment based on sheep found that interference of Sparcl1 increased preadipocyte cell apoptosis, while the overexpression of Sparcl1 could inhibited lipid droplets accumulation and TG content by PPARγ signal pathway (7).

However, population studies on the association of Sparcl1 and metabolic profile and dyslipidemia are lacking. In the current study we observed linear association between serum Sparcl1 and triglycerides level as well as prevalence of dyslipidemia. This is the first study found that serum Sparcl1 was significantly associated with decreased prevalent risk of hypertriglyceridemia and dyslipidemia in Chinese adults, suggesting the importance of Sparcl1 in the metabolic regulation, especially lipid metabolism. Considering the potential effect of sparcl1 on the development of cancer and cardiovascular disease (2, 3, 5), the current study excluded participants with cancer or cardiovascular disease. Therefore, our results on the association of Sparcl1 and dyslipidemia could exclude the influence of these comorbidities to some extent. The results underline the important role of Sparcl1 in the regulation of lipid metabolism. Sparcl1 might be an alternative target of the treatment of hypertriglyceridemia and dyslipidemia. The findings of our population study are exploratory, and may provide potential indication for future researches on the molecular mechanism of the development of dyslipidemia. The potential protective role of Sparcl1 for dyslipidemia needs confirmation in other study populations.

The detailed role and potential mechanisms of Sparcl1 regulate lipid metabolism are not defined. Sparcl1 was previously reported to regulate the PPARγ signaling and the expression of LPL in sheep (7). Interestingly, PPARγ is highly expressed in white adipocyte tissue, and regulates a variety of adipocyte genes involved in lipid metabolism (20, 21). PPARγ increases fatty acid uptake and fatty acid storage in lipid droplets (lipid steal action), thereby decreasing ectopic lipid deposition (22, 23). PPARγ activation by glitazones could reduce insulin resistance and dyslipidemia (22). Besides, LPL is a key enzyme in catabolism of plasma lipoprotein TGs and modulate lipoprotein profiles, especially the plasma levels of triglyceride and HDL-C (24–26). One possibility is that Sparcl1 might regulate the PPARγ signal pathway and LPL expression, thereby regulating lipid metabolism and the development of dyslipidemia. Nevertheless, this hypothesis still needs to be confirmed by further functional studies.

The strengths of the current study include a large community-based cohort, and comprehensive information of lifestyle factors and biochemical indicators. However, some limitations should be indicated. First, the cross-sectional study design cannot assess the causality of serum Sparcl1 and metabolism disorders. Prospective studies are needed to further confirm the association of plasma Sparcl1 and dyslipidemia. Second, our study only included 40 years or older Chinese adults, which would limit the generalizability of our findings. Third, our study participants were relatively healthy. Further studies are warranted to explore the association between serum Sparcl1 and metabolic measures in other populations.

In conclusion, our study found that serum Sparcl1 was inversely associated with the prevalence of dyslipidemia in a Chinese population, suggesting the potential role of Sparcl1 as a metabolic regulator. Experimental and large clinical studies are also warranted to explore the specific role of Sparcl1 in the development of dyslipidemia.

## Data availability statement

The raw data supporting the conclusions of this article will be made available by the authors, without undue reservation.

## Ethics statement

The studies involving human participants were reviewed and approved by The study protocol was approved by the Ethics Committee of Ruijin Hospital, Shanghai Jiao-Tong University School of Medicine. The patients/participants provided their written informed consent to participate in this study.

## Author contributions

JL, YB, WW, and GN conceived and designed the study. Material preparation and data analysis were performed by CH, SW, HQ, and LL. CH, LL, HQ, HL, XJ, YZ, XW, ML, TW, ZZ, MX, YX, MD and YC collected data. The first draft of the manuscript was written by CH and all authors commented on previous versions of the manuscript. JL, YB, WW, and GN are the guarantors of this work and had full access to all the data in the study. All authors contributed to the article and approved the submitted version.

## Funding

This work was supported by the National Natural Science Foundation of China (Grant No. 81970691, 82170819), Shanghai Outstanding Academic Leaders Plan (Grant No. 20XD1422800), Shanghai Medical and Health Development Foundation (Grant No. DMRFP_I_01), Clinical Research Plan of SHDC (Grant No. SHDC2020CR3064B), and Science and Technology Committee of Shanghai (Grant No. 20Y11905100, 19411964200).

## Acknowledgments

The authors thank all participants in the study. We thank Dr Lu Yan for scientific discussion.

## Conflict of interest

The authors declare that the research was conducted in the absence of any commercial or financial relationships that could be construed as a potential conflict of interest.

## Publisher’s note

All claims expressed in this article are solely those of the authors and do not necessarily represent those of their affiliated organizations, or those of the publisher, the editors and the reviewers. Any product that may be evaluated in this article, or claim that may be made by its manufacturer, is not guaranteed or endorsed by the publisher.
